# Inflammatory proteins in maternal plasma, cervicovaginal and amniotic fluids as predictors of intra-amniotic infection in preterm premature rupture of membranes

**DOI:** 10.1371/journal.pone.0200311

**Published:** 2018-07-06

**Authors:** Seung Mi Lee, Kyo Hoon Park, Eun Young Jung, Song Yi Kook, Hyunsoo Park, Se Jeong Jeon

**Affiliations:** 1 Department of Obstetrics and Gynecology, Seoul National University College of Medicine, Seoul, Korea; 2 Department of Obstetrics and Gynecology, Seoul National University Hospital, Seoul, Korea; 3 Department of Obstetrics and Gynecology, Seoul National University Bundang Hospital, Seongnam, Korea; University of Illinois at Urbana-Champaign, UNITED STATES

## Abstract

**Objective:**

We aimed to assess the correlations among multiple cytokine concentrations in the maternal plasma, cervicovaginal fluid (CVF), and amniotic fluid (AF) compartments in women with preterm premature rupture of membranes (pPROM), and to develop a prediction model based on non-invasive measures, having better sensitivity and specificity for the identification of microbial invasion of amniotic cavity (MIAC).

**Method:**

This retrospective study included 75 consecutive women with pPROM (20+0–34+0 weeks), who underwent amniocentesis. Both maternal plasma and CVF samples were collected at the time of amniocentesis. Stored AF, plasma and CVF samples were assayed for cytokine levels [interleukin (IL)-6, IL-8, monocyte chemotactic protein-1, macrophage inflammatory protein (MIP)-1α, MIP-1β] using a multiplex immunoassay kit.

**Results:**

Levels of inflammatory proteins measured in the CVF were significantly correlated with AF proteins levels, whereas none of the proteins in plasma correlated significantly with any in the AF or CVF. Proteins levels measured in the AF and CVF were significantly higher in women with MIAC compared to those without, whereas only high levels of IL-6 in plasma were significantly associated with MIAC. By using stepwise regression analysis, a non-invasive model (using clinical factors and CVF cytokine levels) for the prediction of MIAC was developed; the area under curve of this non-invasive model was similar to that of the invasive model (using clinical factors and AF cytokines).

**Conclusions:**

The levels of inflammatory proteins in the CVF correlated with those in the AF, whereas those in the plasma showed no correlation. A non-invasive model using clinical factors and CVF cytokine levels predicted the risk of MIAC in women with pPROM.

## Introduction

The preterm premature rupture of membranes (pPROM) is a major precursor of spontaneous preterm delivery [1, 2]. The microbial invasion of amniotic cavity (MIAC) is present in about 30–40% of patients with pPROM, and this contributes to an increased risk of preterm birth and adverse neonatal outcomes [3–6]. Therefore, a more precise prediction of MIAC, especially using non-invasive methods, is important for improving pregnancy management and counseling in patients with pPROM.

Traditionally, an algorithm for MIAC prediction has been used which measures inflammatory biomarkers in the amniotic fluid (AF) obtained by amniocentesis, but is at present limited by the requirement of invasive sampling of the AF [6, 7]. Therefore, in recent years research has been focused on the identification of potential biomarkers present in specimens obtained non-invasively [e.g., maternal blood or cervicovaginal fluid (CVF)] that can accurately predict MIAC. However, no biomarkers measured in either the maternal blood or the CVF compartment alone have yet demonstrated to have sufficient sensitivity and specificity to replace the biomarkers obtained from the AF. Moreover, there is little data on whether the expression patterns of multiple inflammatory markers in non-invasive samples (i.e., CVF and maternal blood) are directly correlated with those in AF samples in the setting of MIAC in pPROM [8, 9]. The clarification of these issues is clinically relevant, not only to better understand the relative inflammatory and immune responses to microbes in the different compartments, but also to precisely identify MIAC while avoiding the risk and difficulty of invasive amniocentesis. Only one study has been published on this issue, but it is limited by a small sample size and the fact that not all non-invasive samples were simultaneously assessed in all AF samples [9].

Recently, a multiplex immunoassay has been used in several research areas. This method allows measurement of multiple proteins, and we hypothesized that a combination of multiple biomarkers using both maternal plasma and CVF compartments (non-invasive samples) may increase the diagnostic accuracy of MIAC to clinically useful levels of accuracy with AF testing. In this context, we assessed the correlation among multiple cytokine levels in the maternal plasma, CVF, and AF compartments in women with pPROM, and developed a prediction model based on non-invasive measures, with better sensitivity and specificity for MIAC.

## Materials and methods

### Study population

This retrospective cohort study consisted of consecutive patients who were admitted to the Seoul National University Bundang Hospital with a diagnosis of pPROM (from 21+0 to 34+0 weeks gestation) between November 2008 and July 2014 and fulfilled the following inclusion criteria: 1) singleton pregnancy; 2) trans-abdominal amniocentesis performed to evaluate AF for infection or fetal lung maturity; 3) both maternal blood and CVF samples collected at the time of amniocentesis; and 4) aliquots of plasma, CVF, and AF samples available for analysis. Exclusion criteria were: multiple pregnancies, major congenital anomalies, vaginal bleeding, prior cervical cerclage, active labor (defined by the presence of cervical dilatation >3 cm by sterile speculum examination), and clinical signs of chorioamnionitis. A number of clinical data (but not the laboratory data) contained in this manuscript have been published in Acta Obstet Gynecol Scand (n = 37) and Reproductive Science (n = 58) [8, 10]. The diagnosis of pPROM was made based on the findings of a sterile speculum examination and a combination of pooling of fluid, nitrazine, and ferning tests. During the study period, amniocentesis for the retrieval of AF and CVF and plasma samplings were immediately offered to patients who were admitted with pPROM at our institution. Gestational age was determined based on the last menstrual period and first or second trimester (≤20 weeks) ultrasound results when available. This study was approved by the Institutional Review Board of the Seoul National University Bundang Hospital (project number B-1105/128-102). Patients provided written informed consent for the collection and use of AF, maternal blood, and CVF samples for research purposes. The primary outcome measure was MIAC.

### Sample collection and preparation

After obtaining informed consent, transabdominal amniocentesis was performed under ultrasound guidance. AF was cultured for aerobic/anaerobic bacteria and genital mycoplasmas (*Ureaplasma urealyticum* and *Mycoplasma hominis*) as previously described [11]. The remaining AF was centrifuged at 1500×*g*, 4°C for 10 minutes and aliquoted for storage at -70°C until assay. At the time of amniocentesis, maternal plasma and CVF samples were obtained from all participants. CVF sample collection, processing, and storage have been previously described in detail [10]. Briefly, under sterile speculum examination, CVF samples were collected from the posterior vaginal fornix using sterile Dacron swabs (Puritan Medical, Guilford, ME, USA) placed for 15 seconds to absorb the cervicovaginal secretions. The Dacron swab was transferred to a tube containing 1 mL of sample buffer, and stored at -70°C until further analysis. Maternal peripheral blood samples were collected in EDTA tubes, centrifuged for 10 min, and aliquoted for storage at -70°C until assay. AF, maternal blood, and CVF samples were collected prior to administration of medications such as corticosteroids and antibiotics.

### Inflammatory mediator assays

The AF, plasma, and CVF samples were assayed for pro-inflammatory cytokines [interleukin (IL)-6, IL-8, monocyte chemotactic protein-1 (MCP-1), macrophage inflammatory protein (MIP)-1α, and MIP-1β] using a multiplex immunoassay kit. The assay was performed using a bead-based Bio-Plex Pro™ Assay kit, a custom-designed five-plex cytokine kit, according to the manufacturer's protocol (Bio-Rad Laboratories, Hercules, CA). Prior to the simultaneous measurement of five cytokines, the AF and CVF samples were diluted 1:10 and plasma samples were diluted 1:4. The lower limits of detection for each cytokine in the AF and CVF were as follows: IL-6, 15.5 pg/mL; IL-8, 16.3 pg/mL; MCP-1, 10.4 pg/mL; MIP-1α, 12.0 pg/mL; and MIP-1β, 8.5 pg/mL. The lower limits of detection in the maternal plasma were 9.2 pg/mL for IL-6, 10.0 pg/mL for IL-8, 6.0 pg/mL for MCP-1, 0.2 pg/mL for MIP-1α, and 2.2 pg/mL for MIP-1β. In samples with cytokine levels that were lower than the lowest point on the standard curve, the lowest values of detection were used for analysis. The calculated intra- and inter-assay coefficients of variation were 4.6% and 4.1% for IL-6; 6.7% and 6.9% for IL-8; 6.1% and 6.2% for MCP-1; 6.6% and 7.2% for MIP-1α; and 5.4% and 5.1% for MIP-1β, respectively.

### Management of pPROM and definitions

Management of pPROM has been previously described in detail [5, 10]. MIAC was defined as the presence of a positive AF result for microorganisms. Histologic diagnoses of chorioamnionitis and funisitis were established by the presence of acute inflammation in the amnion, chorion-decidual tissue, chorionic plate, and umbilical cord, according to definitions previously described in detail [12]. Clinical chorioamnionitis was defined according to the criteria proposed by Gibbs et al.[13]: fever (≥37.8°C) and the presence of two or more of the associated clinical findings (uterine tenderness, malodorous vaginal discharge, maternal leukocytosis, maternal tachycardia, and fetal tachycardia).

### Statistical methods

Continuous data were first assessed for normality distribution by the Kolmogorov-Smirnov test and analyzed using Student’s *t*-test and the Mann-Whitney U test, as appropriate. Categorical data were analyzed using the χ^2^ test or Fisher’s exact test. Receiver operating characteristic (ROC) curves for the prediction of MIAC were generated for each cytokine and for clinical variables, and used to determine the best cutoff values for each variable. The optimal cutoff values were chosen based on the Youden index [maximum (sensitivity + specificity)-1]. Each of the variables was dichotomized on the basis of the obtained cutoff value. A multivariate logistic regression analysis with these dichotomous independent variables was then performed using the backward stepwise technique to determine the best combination model for prediction of MIAC. Two separate models were constructed. Model 1 included cytokines in the AF obtained by an invasive technique (amniocentesis) along with clinical demographic variables (invasive model). Model 2 included only non-invasive parameters, such as cytokines in the maternal plasma and CVF, and clinical demographic variables (non-invasive model). Variables with a *P*-value of <0.05 from the univariate analysis were entered into logistic regression, and a *P*-value <0.05 was required for final inclusion in the model. The area under the ROC curve (AUC) was used to assess the discriminatory ability of the model, and the AUCs between models 1 and 2 were compared using the method of DeLong et al [14]. A Spearman’s rank correlation test was used to assess the relationship between various cytokine levels of the AF, plasma, and CVF compartments. All reported *P*-values are two-sided, with a significance level of 0.05. All statistical analyses were conducted with SPSS for Windows version 21.0 (IBM SPSS Statistics, Chicago, IL, USA) and MedCalc Statistical Software version 13.3.1 (MedCalc Software bvba, Ostend, Belgium).

## Results

### Characteristics of the study population

During the study period, a total of 75 women with pPROM met all eligibility criteria and were included in the final analysis. The mean gestational age (± standard deviation [SD]) at sampling was 30.1 ± 3.6 weeks (range, 21.1 to 34.0 weeks). The mean admission (due to pPROM) to amniocentesis interval (± SD) was 5.0 ± 7.7 hours (median, 2.0 hours; range, 0 to 45.0 hours). The prevalence of MIAC was 43.2% (32/74), and the microbes isolated from the AF in 32 cases were *Ureaplasma urealyticum* (n = 22), *Mycoplasma hominis* (n = 19), *Staphylococcus aureus* (n = 2), *Viridans streptococci* (n = 2), *Escherichia coli* (n = 1), *Haemophilus influenzae* (n = 1), *Streptococcus agalactiae* (n = 1), gram-positive cocci (n = 1), and gram-negative cocci (n = 1). Polymicrobial invasion was present in 18 cases [56% (18/32)].

The clinical and demographic characteristics of the study population are described in Table 1. Women with MIAC had a significantly lower median gestational age at sampling and delivered earlier and had a higher incidence rate of histologic chorioamnionitis than those without MIAC.

**Table 1:** Characteristics of the study population according to amniotic fluid culture results.

	Amniotic fluid culture		*P*-value^a^
	Negative (n = 43)	Positive (n = 32)	
Maternal age (years)	33 (21–43)	33 (27–40)	0.472
Nulliparity	54% (23)	41% (13)	0.351
Previous spontaneous preterm birth (<37 weeks)	5% (2)	13% (4)	0.392
Gestational age at sampling (weeks)	32.4 (21.1–34.7)	29.8 (21.1–34.0)	0.002
Admission to sampling interval (hours)	2 (0–25)	3 (0–45)	0.198
Use of tocolytics	54% (23)	53% (17)	1.000
Use of corticosteroids	84% (36)	78% (25)	0.563
Use of antibiotics	95% (41)	100% (32)	0.504
Gestational age at delivery (weeks)^b^ (n = 68)	33.7 (25.7–35.0) (n = 39)	30.4 (21.4–34.1) (n = 29)	<0.001
Sampling-to-delivery interval^b^			
< 2 days	28% (11/39)	48% (14/29)	0.127
< 7 days	51% (20/39)	83% (24/29)	0.010
Histologic chorioamnionitis^b^	33% (13/39)	69% (20/29)	0.004
Funisitis^b^	15% (6/39)	31% (9/29)	0.124

Values are given as the median (range) or % (n).

^a^Using the Mann-Whitney U test, χ^2^ test or Fisher’s exact test as appropriate.

^b^Seven cases were excluded for the analysis because the delivery took place at another institution.

### Inflammatory proteins in maternal plasma, cervicovaginal and amniotic fluids

Table 2 shows the median concentrations, *P*-values, AUC, and best cutoff values for all proteins in the AF, maternal plasma, and CVF in relation to the results of the AF culture. Five proteins in the AF, five proteins in the CVF, and one protein in the maternal plasma had significantly higher concentrations in samples from women with MIAC than in those without MIAC. However, MIP-1α and MIP-1β in the AF and IL-6 in plasma were not detected in significant quantities (undetectable in >10%) and were subsequently excluded from further analysis. As a result, the concentrations of the following eight proteins were significantly higher in cases with MIAC than those without MIAC; IL-6, IL-8, and MCP-1 in AF and IL-6, IL-8, MCP-1, MIP-1α, and MIP-1β in the CVF. The AUCs for these proteins in the AF and CVF ranged from 0.836 to 0.857 and 0.721 to 0.765, respectively.

**Table 2:** Concentrations, areas under the curves, and best cutoff values of MIPs, MCP, and cytokines in amniotic fluid, cervicovaginal fluid, and maternal blood in relation to positive amniotic fluid culture results^a^.

	Amniotic fluid culture		*P*-value^b^	AUC^c^	Cutoff value	<LLOQ
	Negative (n = 43)	Positive (n = 32)				
Amniotic fluid (AF)						
AF IL-6	408.4 (28.5–45372.9)	9932.7 (79.1–157349.5)	**< 0.001**	0.857	1488.7	0%
AF IL-8	256.7 (48.3–16428.9)	5402.2 (51.4–152880.8)	**< 0.001**	0.852	988.46	0%
AF MCP-1	237.8 (10.4–15643.5)	4308.8 (36.9–21194.7)	**< 0.001**	0.836	1166.78	4%
AF MIP-1α	12.0 (12.0–1048.8)	266.1 (12.0–11341.0)	**< 0.001**	0.877	12.7	52%
AF MIP-1β	34.7 (8.5–7529.7)	2647.8 (8.5–23196.9)	**< 0.001**	0.872	310.65	27%
Percentage above LLOQ for AF MIP-1α	18.6% (8)	87.5% (28)	**< 0.001**			
Percentage above LLOQ for AF MIP-1β	58.1% (25)	93.8% (30)	**0.001**			
Cervicovaginal fluid (CVF)						
CVF IL-6	174.5 (15.6–1445.7)	656.9 (15.6–6473.4)	**0.001**	0.732	310.79	3%
CVF IL-8	1451.4 (54.0–25318.7)	6459.1 (778.0–28766.4)	**< 0.001**	0.765	2281.14	0%
CVF MCP-1	446.8 (51.1–7264.9)	1475.3 (142.5–7195.2)	**0.001**	0.721	1341.67	0%
CVF MIP-1α	49.1 (12.1–949.3)	185.3 (12.1–3365.4)	**<0.001**	0.745	124.16	7%
CVF MIP-1β	495.4 (28.2–6583.2)	1480.5 (31.6–7609.3)	**< 0.001**	0.765	990.57	0%
Maternal blood (MB)						
MB IL-6	9.2 (9.2–38.0)	9.2 (9.2–34.4)	**0.004**	0.632	9.16	81%
MB IL-8	10.0 (10.0–77.7)	10.0 (10.0–370.2)	0.799	0.512	11.56	78%
MB MCP-1	31.1 (9.8–137.4)	32.3 (9.8–242.7)	0.932	0.506	25.06	0%
MB MIP-1α	1.0 (0.2–20.4)	0.8 (0.2–9.3)	0.265	0.425	0.77	3%
MB MIP-1β	78.6 (24.5–160.1)	96.4 (20.1–859.1)	0.120	0.605	160.1	0%
Percentage above LLOQ for MB IL-6	7.0% (3)	28.1% (9)	0.023			
Percentage above LLOQ for MB IL-8	20.9% (9)	21.9% (7)	0.921			

Data are given as the median (range) (pg/mL).

AUC, areas under the curves; LLOQ, lower limit of quantification; AF, amniotic fluid; CVF, cervicovaginal fluid; MB, maternal blood; IL, interleukin; MCP-1, monocyte chemotactic protein-1; MIP, macrophage inflammatory protein.

^a^Significant findings (*p* < 0.01) after Bonferroni correction for multiple testing (α threshold = 0.05/5) are presented in bold letters.

^b^Mann-Whitney U test.

^c^Receiver operating characteristic curve analysis.

Table 3 shows the correlations among protein levels in the AF, CVF, and maternal plasma. Only proteins detected in significant quantities (>90%) are shown. There was a high positive correlation between protein levels in the CVF and AF compartments. However, none of the proteins in the maternal plasma were significantly correlated with those in the AF or CVF compartments. Within the AF and CVF compartments, protein levels were highly correlated, whereas within the maternal plasma, only MCP-1 and MIP-1β levels showed significant correlation.

**Table 3:** Pairwise Spearman’s rank correlation coefficients for MIP, MCP, and cytokine concentrations in the amniotic fluid, cervicovaginal fluid, and maternal blood.

	AF IL-6	AF IL-8	AF MCP-1	CVF IL-6	CVF IL-8	CVF MCP-1	CVF MIP-1α	CVF MIP-1β	MB MCP-1	MB MIP-1α
Amniotic fluid (AF)										
AF IL-8	0.955 ^b^									
AF MCP-1	0.913 ^b^	0.902 ^b^								
Cervicovaginal fluid (CVF)										
CVF IL-6	0.413 ^b^	0.416 ^b^	0.398 ^b^							
CVF IL-8	0.555 ^b^	0.566 ^b^	0.531 ^b^	0.474^b^						
CVF MCP-1	0.569 ^b^	0.588 ^b^	0.615 ^b^	0.739^b^	0.599 ^b^					
CVF MIP-1α	0.515 ^b^	0.514 ^b^	0.502 ^b^	0.690^b^	0.764 ^b^	0.809 ^b^				
CVF MIP-1β	0.575 ^b^	0.550 ^b^	0.525 ^b^	0.711^b^	0.710 ^b^	0.772 ^b^	0.940 ^b^			
Maternal blood (MB)										
MB MCP-1	0.011	-0.021	0.063	0.015	-0.020	0.076	0.024	0.046		
MB MIP-1α	-0.204	-0.208	-0.161	-0.018	-0.083	-0.097	-0.055	-0.038	-0.170	
MB MIP-1β	0.098	0.005	0.020	0.025	-0.065	0.034	0.073	0.180	0.324^a^	0.172

AF, amniotic fluid; CVF, cervicovaginal fluid; MB, maternal blood; IL, interleukin; MCP-1, monocyte chemotactic protein-1; MIP, macrophage inflammatory protein.

^a^ p < 0.01.

^b^ p < 0.001.

### Development of a prediction model for MIAC

To develop the best prediction model for MIAC, cytokine levels in the AF, CVF, and maternal plasma along with clinical parameters were included in the multivariate analysis. Among clinical factors, only gestational age at sampling was included. The variables entered in the multivariate analysis were selected based on a *P*-value of <0.05 in univariate analyses and all continuous parameters were dichotomized using the cutoff values derived from the ROC curves.

In model 1 (invasive model), only high AF levels of IL-6 (>1488.7 pg/mL) and high AF levels of IL-8 (>988.46 pg/mL) were retained in the best prediction model (Table 4). The Hosmer-Lemeshow test showed a *P*-value of 0.182 indicating an adequate model fit. In model 2 (non-invasive model), proteins in the CVF along with baseline variables were also included in the multivariate analysis. The final variables retained in the non-invasive model were earlier gestational age at sampling (≤31.4 weeks) and high levels of IL-6 (>310.79 pg/mL) in the CVF (Table 4). The Hosmer-Lemeshow test showed a *P*-value of 0.779 indicating an adequate model fit.

**Table 4:** Regression coefficients, odds ratios, and 95% confidence intervals of the final combined model for predicting positive AF culture among 75 women with preterm premature rupture of membranes.

Predictor	Beta-coefficient	SE	OR (95% CI)	*p*-value
**Invasive model**				
High AF IL-6 level (> 1488.7 pg/mL)	2.067	0.928	7.903 (1.282–48.707)	0.026
High AF IL-8 level (> 988.46 pg/mL)	1.790	0.907	5.987 (1.011–35.446)	0.049
Constant	-2.335	0.553	0.097	< 0.001
**Non-invasive model**				
Earlier GA at sampling (≤ 31.4 weeks)	2.132	0.637	8.430 (2.417–29.399)	0.001
High CVF IL-6 level (> 310.79 pg/mL)	2.245	0.629	9.443 (2.750–32.425)	<0.001
Constant	-2.528	0.633	0.080	< 0.001

SE, standard error; OR, odds ratio; CI, confidence interval; AF, amniotic fluid; IL, interleukin; GA, gestational age; CVF, cervicovaginal fluid

**Fig 1:**
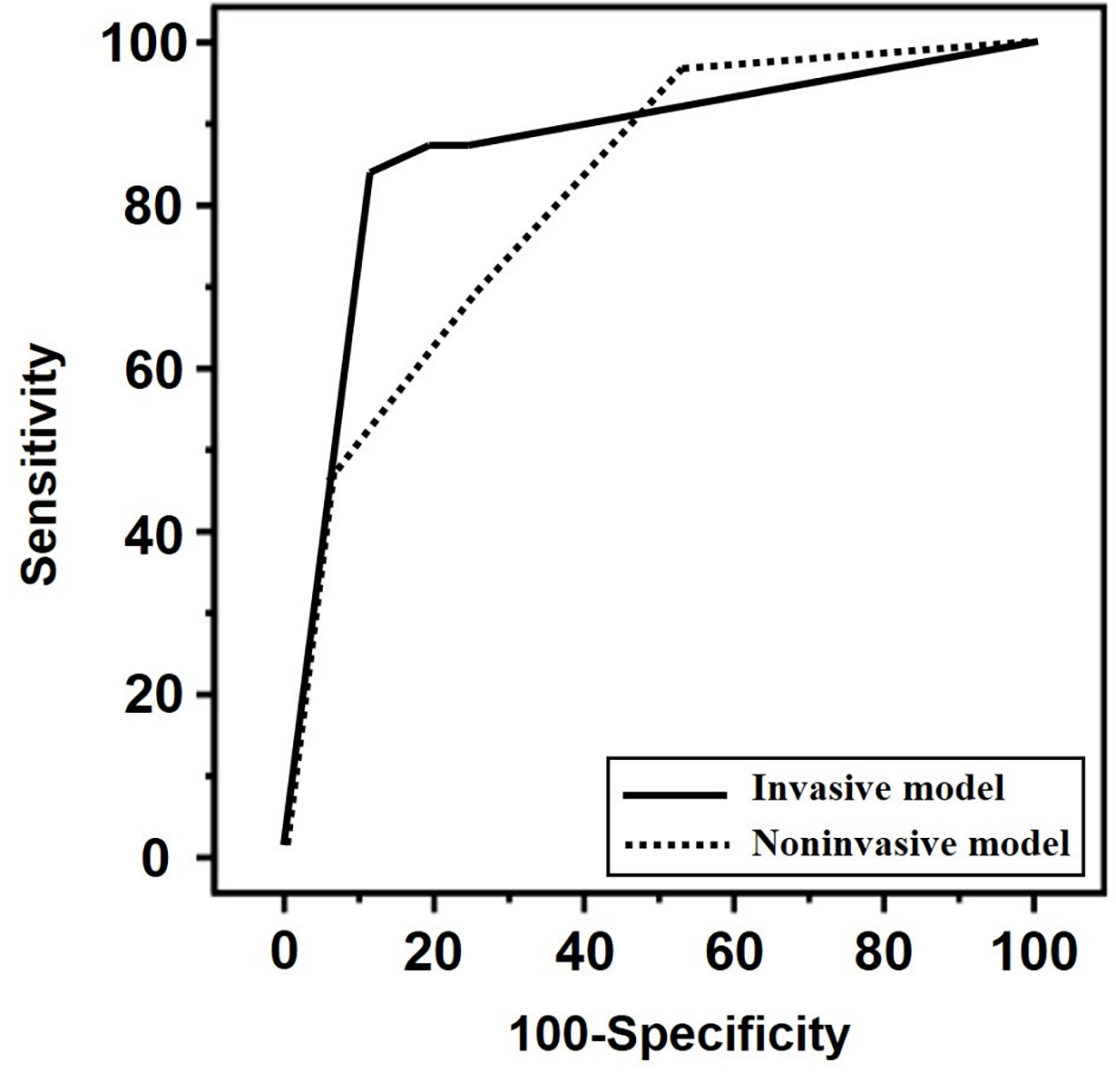
Receiver operating characteristics (ROC) curves for the invasive model (solid line) and non-invasive combined model (dotted line) in predicting microbial invasion of amniotic cavity (invasive model: area under the curve [AUC], 0.869; 95% confidence interval [CI], 0.771–0.936 versus non-invasive model: AUC, 0.813; 95% CI, 0.706–0.936; *P* = 0.253 for testing two ROC curves).

Fig 1 compares the ROC curves of the non-invasive and invasive models. The AUC of the non-invasive model was similar to that of the invasive model (*P* = 0.253). The AUC of invasive model was 0.869 [95% confidence interval (CI), 0.771–0.936] and that of non-invasive model was 0.813 [95% CI, 0.706–0.936].

## Discussion

The principal findings of this study are as follows: (1) in women with pPROM, the levels of inflammatory proteins measured in the CVF were significantly correlated with protein levels in the AF, whereas none of the proteins in the maternal plasma correlated significantly with any in the AF or CVF; (2) protein levels measured in the AF and CVF were significantly higher in women with MIAC compared to those without, whereas only high levels of IL-6 in the maternal plasma showed a significant association with MIAC; and (3) the non-invasive model using clinical factors and CVF proteins was as accurate as the invasive model based on clinical variables and AF protein levels to predict the occurrence of MIAC. The latter is particularly relevant in a clinical perspective given the non-invasiveness of measuring biomarkers in the CVF and maternal plasma samples.

This study is, to our knowledge, the first to directly compare inflammatory protein levels in the plasma, CVF, and AF compartments in relation to MIAC in women with pPROM [Searches of PubMed (January 1966 through February 2018), EMBASE (January 1966 through February 2018), and the Cochrane Library were conducted using the following search terms: ‘preterm premature rupture of membranes’, ‘intraamniotic infection’, ‘amniotic fluid’, ‘blood’, and ‘cervicovaginal fluid’ or ‘cervical fluid’ or ‘vaginal fluid’]. We found that the levels of inflammatory proteins measured in the CVF were significantly correlated with protein levels in the AF, whereas protein levels in the maternal plasma showed no correlation. Similarly, in the current study, inflammatory proteins in the CVF were elevated in the presence of MIAC, which is in line with previously published data in pPROM cases [10, 15–17]. However, we did not find different expression levels of inflammatory proteins (except for IL-6) in response to MIAC in the maternal plasma compartment. Collectively, these findings suggest that the potential use of CVF samples for non-invasive quantification of proteins for predicting MIAC is greater than that of maternal plasma in women with pPROM. In fact, this result is plausible considering that in pPROM small quantities of AF may spill into the vagina, thus the characteristics of the CVF mixed with AF leaked into the vagina may be similar to those of AF in the amniotic cavity. Contrary to the CVF samples, plasma is located in an area that is distal to the original site (i.e., the AF), where the host immune response to microorganisms occurs. Thus, it is particularly difficult for the maternal immune system in plasma to respond to either microbes or pro-inflammatory mediators produced in the amniotic cavity. Accordingly, future studies should utilize CVF samples, rather than those of maternal blood to identify novel non-invasive biomarkers for MIAC. In this regard, standardized methods to process CVF samples may be important for the assessment of MIAC. For example, in contrast to the current study, Cobo et al. did not find any significant elevation of inflammatory protein levels in the vaginal fluid (except for C-reactive protein) in the presence of MIAC [9]. Sampling methods for CVF may be a contributing factor for this discrepancy between our findings and those of Cobo et al [9]. In the study by Cobo et al., vaginal mucus samples were obtained by Cytobrush swab [9], whereas cervicovaginal secretions at the posterior vaginal fornix were collected using the Dacron swab in the present study.

In addition, our finding that the diagnostic accuracy of the noninvasive model was similar to that of the invasive model in terms of MIAC differentiation is the first report that compares efficacy of non-invasive and invasive models for identifying MIAC [Searches of PubMed (January 1966 through February 2018), EMBASE (January 1966 through February 2018), and the Cochrane Library were conducted using the following search terms: ‘noninvasive model’, ‘invasive model’, ‘preterm premature rupture of membranes’, ‘intraamniotic infection’ or ‘microbial invasion of amniotic cavity’]. Similar observations have also been reported in studies involving histologic chorioamnionitis and imminent preterm birth in women with pPROM by our group [5, 18]. From a clinical perspective, our non-invasive model can be used as a screening method for MIAC rather than a diagnostic tool as the rational choice for antibiotic selection requires the identification of specific microorganisms through AF studies [19]. Moreover, this non-invasive model is also useful for predicting MIAC in women in whom amniocentesis is not possible due to oligohydramnios [20, 21]. Further studies are needed to examine whether this non-invasive model is useful for serial evaluation of patients to determine the response to the antibiotic therapy or to check for a possible ascending infection after the rupture of membranes.

In the current study, among the biomarkers measured in the maternal plasma, only the median IL-6 level differed significantly according to the presence or absence of MIAC, although it was not detected in significant quantities, which suggests a weak maternal inflammatory response in the plasma to MIAC. Similarly, Cobo et al. reported that a higher but weak expression of maternal serum IL-6 (20.8 vs. 13.9 pg/mL, *P* = 0.019), which was the only serum biomarker for MIAC, was observed in women with preterm labor (22+0 to 31+6 weeks of gestation) who had MIAC [22]. Taken together, the findings of our group and those of Cobo et al [22]. suggest that the status of the amniotic cavity may not be accurately reflected in the maternal blood and thus the assessment of inflammatory proteins in the maternal blood may be of limited clinical usefulness for identifying MIAC.

Consistent with the findings from previous studies on women with preterm labor with or without membrane rupture [8, 9, 23], a more significant inflammatory response in the AF is observed in the presence of MIAC compared with the CVF or maternal plasma (Table 2). These findings are not unexpected because the chemical mediators produced by microorganisms in the amniotic cavity are usually released more directly and intensely at the site of infection (i.e., AF), where the host immune response to microbial pathogens occurs, rather than in any site distant from the microorganisms.

The main strength of the current study is that we obtained samples from the AF, the maternal plasma, and the CVF simultaneously, which allowed for a direct comparison of the cytokine levels in these three compartments. This strengthens the evidence from the current study indicating that CVF cytokines are better markers than plasma cytokines for evaluating the infectious/inflammatory status in the AF. However, the major limitations of the current study include its retrospective nature, its use of a relatively small sample size from a single hospital, limited inflammatory protein assessment, and that the prediction models were not validated with test samples, which may limit the generalizability of the study findings. Larger prospective studies are needed to validate our non-invasive model in other study populations. Furthermore, this study was limited by relying on the use of traditional culture techniques to identify bacteria from the AF, and the lack of application of more sensitive molecular techniques (i.e., polymerase chain reaction) for determining the presence of bacteria in the samples. This cannot exclude the possibility of the presence of uncultivated microorganisms in the women with intra-amniotic infection. A further limitation was that we were unable to assay other inflammatory proteins that are generally considered to be the most relevant in intra-amniotic infections, such as IL-1β, IL-10, IL-18, and MMPs, because of limited research funding available [22–24]. Lastly, for the current study, the candidate proteins evaluated were selected because they had previously been associated with the presence of MIAC in several studies based on samples obtained from the plasma, CVF, or AF of women with pPROM and preterm labor [8, 9, 22, 23, 25].

## Conclusions

In conclusion, our study reveals that the levels of inflammatory proteins measured in the CVF were significantly correlated with protein levels in the AF, whereas none of the proteins in the maternal plasma correlated significantly with any in the AF or CVF. Furthermore, we have developed a non-invasive model using clinical factors and CVF cytokine concentrations that acts a good predictor of the risk of MIAC in women with pPROM. However, the assessment of inflammatory markers in the maternal plasma may be of limited clinical usefulness for the identification of MIAC in this setting.

## Supporting information

S1 File Raw data. (SAV)

## References

[1] Goldenberg RL, Culhane JF, Iams JD, Romero R. Epidemiology and causes of preterm birth. Lancet. 2008;371(9606):75–84. doi: 10.1016/S0140-6736(08)60074-4 . 18177778 10.1016/S0140-6736(08)60074-4 PMC7134569

[2] Kaltreider DF, Kohl S. Epidemiology of preterm delivery. Clin Obstet Gynecol. 1980;23(1):17–31. . 6988128 10.1097/00003081-198003000-00005

[3] Goncalves LF, Chaiworapongsa T, Romero R. Intrauterine infection and prematurity. Ment Retard Dev Disabil Res Rev. 2002;8(1):3–13. doi: 10.1002/mrdd.10008 . 11921380 10.1002/mrdd.10008

[4] Berger A, Witt A, Haiden N, Kaider A, Klebermasz K, Fuiko R, et al Intrauterine infection with Ureaplasma species is associated with adverse neuromotor outcome at 1 and 2 years adjusted age in preterm infants. J Perinat Med. 2009;37(1):72–8. doi: 10.1515/JPM.2009.016 . 18976044 10.1515/JPM.2009.016

[5] Park KH, Lee SY, Kim SN, Jeong EH, Oh KJ, Ryu A. Prediction of imminent preterm delivery in women with preterm premature rupture of membranes. J Perinat Med. 2012;40(2):151–7. doi: 10.1515/JPM.2011.124 . 22085152 10.1515/JPM.2011.124

[6] Shim SS, Romero R, Hong JS, Park CW, Jun JK, Kim BI, et al Clinical significance of intra-amniotic inflammation in patients with preterm premature rupture of membranes. Am J Obstet Gynecol. 2004;191(4):1339–45. doi: S0002937804007082. doi: 10.1016/j.ajog.2004.06.085 . 15507963 10.1016/j.ajog.2004.06.085

[7] Romero R, Yoon BH, Mazor M, Gomez R, Gonzalez R, Diamond MP, et al A comparative study of the diagnostic performance of amniotic fluid glucose, white blood cell count, interleukin-6, and gram stain in the detection of microbial invasion in patients with preterm premature rupture of membranes. Am J Obstet Gynecol. 1993;169(4):839–51. . 7694463 10.1016/0002-9378(93)90014-a

[8] Jung EY, Park KH, Han BR, Cho SH, Ryu A. Measurement of interleukin 8 in cervicovaginal fluid in women with preterm premature rupture of membranes: a comparison of amniotic fluid samples. Reprod Sci. 2017;24(1):142–7. doi: 10.1177/1933719116651149 . 27233755 10.1177/1933719116651149

[9] Cobo T, Jacobsson B, Kacerovsky M, Hougaard DM, Skogstrand K, Gratacos E, et al Systemic and local inflammatory response in women with preterm prelabor rupture of membranes. PLoS One. 2014;9(1):e85277 doi: 10.1371/journal.pone.0085277 . 24465522 10.1371/journal.pone.0085277 PMC3897420

[10] Ryu A, Park KH, Oh KJ, Lee SY, Jeong EH, Park JW. Predictive value of combined cervicovaginal cytokines and gestational age at sampling for intra-amniotic infection in preterm premature rupture of membranes. Acta Obstet Gynecol Scand. 2013;92(5):517–24. doi: 10.1111/aogs.12073 23324124 10.1111/aogs.12073

[11] Lee SY, Park KH, Jeong EH, Oh KJ, Ryu A, Kim A. Intra-amniotic infection/inflammation as a risk factor for subsequent ruptured membranes after clinically indicated amniocentesis in preterm labor. J Korean Med Sci. 2013;28(8):1226–32. doi: 10.3346/jkms.2013.28.8.1226 . 23960452 10.3346/jkms.2013.28.8.1226 PMC3744713

[12] Yoon BH, Romero R, Kim CJ, Jun JK, Gomez R, Choi JH, et al Amniotic fluid interleukin-6: a sensitive test for antenatal diagnosis of acute inflammatory lesions of preterm placenta and prediction of perinatal morbidity. Am J Obstet Gynecol. 1995;172(3):960–70. . 7892891 10.1016/0002-9378(95)90028-4

[13] Gibbs RS, Blanco JD, St Clair PJ, Castaneda YS. Quantitative bacteriology of amniotic fluid from women with clinical intraamniotic infection at term. J Infect Dis. 1982;145(1):1–8. . 7033397 10.1093/infdis/145.1.1

[14] DeLong ER, DeLong DM, Clarke-Pearson DL. Comparing the areas under two or more correlated receiver operating characteristic curves: a nonparametric approach. Biometrics. 1988;44(3):837–45. . 3203132

[15] Jun JK, Yoon BH, Romero R, Kim M, Moon JB, Ki SH, et al Interleukin 6 determinations in cervical fluid have diagnostic and prognostic value in preterm premature rupture of membranes. Am J Obstet Gynecol. 2000;183(4):868–73. doi: S0002-9378(00)74795-8 [pii] doi: 10.1067/mob.2000.109034 . 11035328 10.1067/mob.2000.109034

[16] Rizzo G, Capponi A, Vlachopoulou A, Angelini E, Grassi C, Romanini C. Interleukin-6 concentrations in cervical secretions in the prediction of intrauterine infection in preterm premature rupture of the membranes. Gynecol Obstet Invest. 1998;46(2):91–5. doi: goi46091 [pii]. doi: 10.1159/000010009 . 9701687 10.1159/000010009

[17] Kayem G, Goffinet F, Batteux F, Jarreau PH, Weill B, Cabrol D. Detection of interleukin-6 in vaginal secretions of women with preterm premature rupture of membranes and its association with neonatal infection: a rapid immunochromatographic test. Am J Obstet Gynecol. 2005;192(1):140–5. doi: S0002937804007653 [pii] doi: 10.1016/j.ajog.2004.07.015 . 15672016 10.1016/j.ajog.2004.07.015

[18] Kim SA, Park KH, Lee SM. Non-Invasive Prediction of Histologic Chorioamnionitis in Women with Preterm Premature Rupture of Membranes. Yonsei medical journal. 2016;57(2):461–8. doi: 10.3349/ymj.2016.57.2.461 . 26847301 10.3349/ymj.2016.57.2.461 PMC4740541

[19] Leekha S, Terrell CL, Edson RS. General principles of antimicrobial therapy. Mayo Clin Proc. 2011;86(2):156–67. doi: 10.4065/mcp.2010.0639 . 21282489 10.4065/mcp.2010.0639 PMC3031442

[20] Locatelli A, Ghidini A, Verderio M, Andreani M, Strobelt N, Pezzullo J, et al Predictors of perinatal survival in a cohort of pregnancies with severe oligohydramnios due to premature rupture of membranes at <26 weeks managed with serial amnioinfusions. Eur J Obstet Gynecol Reprod Biol. 2006;128(1–2):97–102. doi: 10.1016/j.ejogrb.2006.02.003 . 16530921 10.1016/j.ejogrb.2006.02.003

[21] Kacerovsky M, Musilova I, Andrys C, Drahosova M, Hornychova H, Rezac A, et al Oligohydramnios in women with preterm prelabor rupture of membranes and adverse pregnancy and neonatal outcomes. PLoS One. 2014;9(8):e105882 doi: 10.1371/journal.pone.0105882 . 25171293 10.1371/journal.pone.0105882 PMC4149497

[22] Cobo T, Tsiartas P, Kacerovsky M, Holst RM, Hougaard DM, Skogstrand K, et al Maternal inflammatory response to microbial invasion of the amniotic cavity: analyses of multiple proteins in the maternal serum. Acta Obstet Gynecol Scand. 2013;92(1):61–8. doi: 10.1111/aogs.12028 . 23057959 10.1111/aogs.12028

[23] Holst RM, Hagberg H, Wennerholm UB, Skogstrand K, Thorsen P, Jacobsson B. Prediction of microbial invasion of the amniotic cavity in women with preterm labour: analysis of multiple proteins in amniotic and cervical fluids. BJOG. 2011;118(2):240–9. doi: 10.1111/j.1471-0528.2010.02765.x . 21054762 10.1111/j.1471-0528.2010.02765.x

[24] Oh KJ, Park KH, Kim SN, Jeong EH, Lee SY, Yoon HY. Predictive value of intra-amniotic and serum markers for inflammatory lesions of preterm placenta. Placenta. 2011;32(10):732–6. doi: 10.1016/j.placenta.2011.07.080 . 21839511 10.1016/j.placenta.2011.07.080

[25] Combs CA, Garite TJ, Lapidus JA, Lapointe JP, Gravett M, Rael J, et al Detection of microbial invasion of the amniotic cavity by analysis of cervicovaginal proteins in women with preterm labor and intact membranes. Am J Obstet Gynecol. 2015;212(4):482 e1–e12. doi: 10.1016/j.ajog.2015.02.007 . 25687566 10.1016/j.ajog.2015.02.007

